# Determinants of pain in advanced HCC patients recieving hepatic artery infusion chemotherapy

**DOI:** 10.1007/s10637-020-01009-x

**Published:** 2020-10-01

**Authors:** Zhiqiang Wu, Wenbo Guo, Song Chen, Wenquan Zhuang

**Affiliations:** Department of Interventional Radiology, the First Affiliated Hospital of Sun Yat-Sen University, No. 58 Zhongshan 2 Road, Guangzhou, 510080 China

**Keywords:** HCC, HAIC, VAS score, Pain, Oxaliplatin, Lidocaine

## Abstract

*Purpose* Hepatic arterial infusion chemotherapy (HAIC) is one of the options to treat unresectable hepatocellular carcinoma (HCC). The majority of HCC patients suffer great pain in the course of HAIC treatment. To improve the quality of life and the efficacy of HAIC treatment, the causes of pain, the choice of an analgesic regimen, and the relationship between pain and prognosis of HCC were analyzed. *Methods* A total of 376 HCC patients under HAIC in our hospital were recriuted between March 2017 and September 2019. Multivariate linear regression analysis (stepwise) was used to calculate the potential factors related to the severe pain in HCC patients under HAIC. Analgesics treatments were carried out based on the results of the visual analogue scale (VAS) score which was used to evaluate the pain. *Results* The mean value of the VAS score is 3.604, which indicates that the pain in most patients is mild and endurable. Intra-arterial lidocaine injection is an effective method in most patients (96%, 361 of 376), and the total score of VAS is reduced from 1355 to 195 following lidocaine injection. Multivariate analysis suggestes that oxaliplatin (OXA) preparation time, hepatic artery diameter and OXA manufacturers (R^2^ = 0.859) are influential factors for pain scores. *Conclusion* This study demonstrates an effective way to systematically assess and ease pain in HCC patients with HAIC treatment. OXA preparation time, hepatic artery diameter, and OXA manufacturers are the potential influencing factors for pain. This work presented here will provide a detailed understanding of the clinical application of HAIC in advanced HCC patients.

## Introduction

Primary hepatocellular carcinoma (HCC) is the fourth leading cause of cancer death in the world [1]. About 25% to 70% of patients were diagonized with advanced liver cancer at the time of diagnosis, and the median survival time of HCC patients is only 4.2 to 7.9 months [2]. The incidence of liver cancer will continue to increase in the next 10 to 20 years, and reach its peak around 2030 [3]. At present, the main treatment for HCC is radical surgical resection, but the recurrence rate after radical resection is still high. Meanwhile, the long-term efficacy of this disease is still not ideal [4]. The recurrence rate within 5 years after the radical operation is as high as 60% [5], and about 90% of liver cancer recurrences occur in the liver [6]. From a clinical perspective, tumor diameter, number, and presence or absence of microscopic tumor emboli are currently recognized as major risk factors which can affect postoperative recurrence [7]. HCC patients often suffer from a variety of adverse factors, such as a large tumor volume, multiple cancer foci, and microscopic tumor emboli. Although the tumor is completely removed, early recurrence after surgery may also be caused due to the formation of microsatellite lesions before surgery, shedding of tumor plugs during surgery, residual cancer cells and body immunosuppression after surgery [8–10]. Therefore, how to suppress or eliminate high-risk factors after surgery is the key to improve the long-term survival rate of HCC patients [11].

The blood supply of HCC and liver metastases is mainly from the hepatic artery, while the blood in normal liver cells are is mainly supplied by the portal vein [12]. Hepatic arterial infusion chemotherapy (HAIC) has been widely used in the treatment of a variety of liver malignancies due to its unique pharmacokinetic advantages: 1) liver cancer tissues can take more chemotherapeutic drugs than systemic chemotherapy. For chemotherapy drugs with steep dose-response curves, it can significantly improve the efficacy of chemotherapy; 2) HAIC can reduce the number of chemotherapeutic drugs to enter the systemic circulation. Thus, the systemic toxicity of the drug is also reduced [13]. 3) HAIC can bring a higher disease remission rate compared with intravenous chemotherapy. For example, Wang et al. showed that the total effective rate of oxaliplatin (OXA) combined with fluorouracil (FU) via HAIC for advanced cholangiocarcinoma is 67.6% [14]. Zhao et al. suggested that HAIC of OXA and FU treatment of advanced primary liver cancer has a median progression-free survival time of 6.1 months. Partial response (PR) is achieved in 28.6% of patients. Furthermore, the incidence of neurotoxicity of the combined treatment regimen is low and the patient’s clinical compliance is strong [15]. Moreover, in advanced HCC patients with portal vein tumor thrombosis (PVTT), HAIC combined with chemotherapeutic drugs is superior to sorafenib in the median time to-progression (TTP) [16]. Also, HAIC is relatively simple and can be repeatedly perfused, which can increase the local drug concentration and reduce systemic side effects. Although 5-FU and cisplatin-based schemes are currently used, the best scheme is still unclear.

HAIC has been reported to be safe and effective for treating unresectable advanced HCC. However, clinical studies found that some patients experienced severe pain during arterial chemotherapy, and sometimes even reached visual analogue scale (VAS)10, leading to a significant reduction in patient tolerance and compliance with this treatment regimen. To improve patients’ quality of life and treatment efficacy of HAIC, this study collected clinical data of 376 patients with HAIC in our hospital from March 2017 to September 2019, and evaluated the causes of pain, the choice of an analgesic regimen, and the relationship between pain and prognosis of HCC.

## Materials and method

### Patients

From March 2017 to September 2019, a total of 376 patients with primary HCC were confirmed by radical surgery and pathology in our hospital. This study was approved by the Ethics Committee of the First Affiliated Hospital of Sun Yat-sen University. Patients in this study received necessary antiviral (Entecavir) and immune (Thymopeptide) treatments after surgery. The clinical data were collected including age, gender, hepatitis status, OXA manufacturers, B lymphoblastoid cell line (BLCL) staging, Child-Pugh score classification, analgesic measures, catheterized hepatic artery diameter, OXA preparation time (formulation to use), intrinsic hepatic artery diameter, microcatheter, visual analogue scale (VAS) score, pain level, lidocaine in arteries, effectiveness of lidocaine, and valid N (listwise).

Inclusion criteria: 1) Age ≤ 75 years; 2) Primary HCC was confirmed by radical surgery and pathology; 3) Child function grade A to B; 4) No other anti-tumor treatment; 5) Survival time > 3 Month; 6) Written consent was obtained; 7) Patients with no pathology needed to meet the criteria for diagnosis and treatment of primary liver cancer (2017 Edition); (8) All patients had not received any form of anti-tumor treatment before their visit to our hospital.

Exclusion criteria: 1) Patients with cholangiocarcinoma after surgery or puncture; 2) Patients with a second primary tumor other than HCC.

### HAIC method

The femoral artery was intubated by the Seldinger method for 3 weeks after the operation. The catheter was sent to the hepatic artery or left and right hepatic artery for angiography. After the fixation of the catheter, 85 mg/m^2^ OXA was injected through the hepatic artery. A total of 2 courses of treatment were carried out (one course every 4 weeks).

### VAS scoring system for the pain of cancer patients

VAS scoring system included: 1) Homemade measuring ruler: a cardboard with a length of 10 cm and a width of about two horizontal fingers was chosen. A layer of white, slightly hard paper had no scale on one side, and only a black straight line was drawn in the middle. There was a scale of 0 to 10 on the other side: 0 on one end meant no pain. 10 on the other end meant severe pain, and middle part for different levels of pain: 1 to 3 pain was mild, and could be tolerated. Sleep was not affected; 4 to 6 pain affected sleep, tolerable; 7 to 9 represented the gradually increasing pain, unbearable; 10 meant severe pain. 2) Self-made facial expression chart for cancer pain assessment: based on the VAS measuring ruler, the facial expression chart of paint was pasted on cardboard of corresponding length and width. On the other side was a scale engraved with 0 to 10. It was suitable for the elderly, children, patients with low education levels, patients with impaired expression and patients with cognitive impairment. 3) Evaluation: in clinic, the graduated side of the measuring ruler was turn back to the patient, and the straight line was drawn where the patient facedd. A point was marked on the straight line that represented the degree of pain, and the patient’s pain intensity score was on the back according to the corresponding scale. The facial expressions were used in the same way as the VAS measuring ruler.

### Pain treatments

In this study, different measures were taken according to different VAS scores: 0 meant no analgesic measures; 1 meant the use of weak opioids for analgesia; 2 meant the use of strong opioids for analgesia; and 3 meant the use of enhanced opiates Analgesic. The evaluation of the analgesic effect was performed according to the following criteria: ineffective; effective indicated that the pain score decreased by more than 3 points; and obviously effective meant that the pain score was decreased by more than 6 points.

### Statistical processing

Statistical Product and Service Solutions (SPSS) version 22.0 statistical software (SPSS Inc., Chicago, IL, USA) and R software (http://www.R-project.org) were used for statistical analysis. The data that conformed to the normal distribution were described by mean ± standard deviation (SD). Counting data are described as percentages. The stepwise method was used in this study. *P* < 0.05 was considered statistically significant.

## Results

### Clinical information of all patients

In this study, all clinical information of 376 patients was collected, including age, gender, hepatitis status, OXA manufacturers, BLCL staging, Child-Pugh score classification, analgesic measures, catheterized hepatic artery diameter, OXA preparation time, intrinsic hepatic artery diameter, microcatheter, VAS score, pain level, lidocaine in arteries, effectiveness of lidocaine, and valid N (listwise). As shown in Table 1, 376 patients included 88.8% males and 11.2% females with mean age 50.80 ± 12.18 years. VAS score of all patients was 3.60 ± 3.36, indicating that the pain in most of the patients was mild and tolerable, and sleep was not affected. Moreover, pain level analysis revealed that the mean value of all patients was 1.32, which was consistent with the VAS score system. Catheter arterial diameter of patients was 2.67 ± 0.93 mm, and 48% patients used microcatheter. OXA used in this study was 60% imported and 40% domestic. 59% OXA time (from formulation to use) was less than 4 h and 41% was more than 4 h. It was also noticed that intra-arterial lidocaine injection was effective in most patients (96%, 361 of 376).

**Table 1:** Basic information of all patients participated in this study

Items	Minimum	Maximum	x ± SD
Gender	Male(88.8%)	Female(11.2%)	
Age	22	80	50.80 ± 12.18
Hepatitis (0 = none, 1 = yes)	Yes (91%)	No(9%)	
OXA manufacturers (0 = import, 1 = domestic)	Import (60%)	Domestic (40%)	
BLCL staging (1 = B stage, 0 = C period)	B(14%)	C(86%)	
Child Pugh classification (0 = A level, 1 = B level, 2 = C level)	A(88%)	B(12%)	
Catheter arterial diameter(mm)	1	4.5	2.67 ± 0.93
OXA preparation time	Less than 4 h (59%)	More than 4 h (41%)	
Hepatic artery diameter (mm)	2.7	4.5	3.50 ± 0.52
Useage of Microcatheter	No(52%)	Yes(48%)	
VAS score	0	10	3.60 ± 3.36
Useage of lidocaine	No (57%)	Yes (43%)	
Effectivity of lidocaine	No effectivity (4%)	Effectivity (96%)	

### Pain levels and analgesics

In this study, we divided all patients into 4 different groups based on the pain levels. For pain level = 0, there were 134 patients (35.6%). For pain level = 1, there were 72 patients (19.1%). For pain level = 2, there were 85 patients (22.6%). For pain level = 3, there were 85 patients (22.6%). More than half of patients had pain levels less than or equal to 1. However, it was worth noting that 45.2% (170) of patients were suffering from a pain index greater than or equal to 2 (Table 2).

**Table 2:** Grade of pain levels of patients in this study

Grade of Pain	Frequency	Percent
0	134	35.6%
1	72	19.1%
2	85	22.6%
3	85	22.6%
Total	376	100%

Meanwhile, the analgesics treatments for patients with different pain levels were evaluated. Results in Table 3 showed that no analgesia patients (36.2%, 136 of 376) and strong opioid analgesics patients (43.9%, 165 of 376) accounted for the largest proportion. Less than 40% of patients did not need analgesics, and less than half of patients required strong opioid analgesics.

**Table 3:** Analgesic treatment for patients with different pain degree

Classifications of treatment	Frequency	Percentage
A	136	36.2%
B	69	18.4%
C	165	43.9%
D	6	1.6%
Total	376	100.0%

Classification A = no analgesia, B = weak opioid, C = strong opioid, D = additional morphine

### BLCL stage and child-Pugh stage

In this study, the BLCL stage (Table 4) and Child-Pugh stage (Table 5) were applied to distinguish HCC patients at different stages. The results suggested that these two classification methods obtained similar results. In all patients, 85.6% (322 of 376) patients were in BLCL C stage and 14.4% (54 of 376) patients were in B stage. In addition, 88.3% (332 of 376) patients belonged to Child-Pugh A stage, and 11.7% (44 of 376) patients belonged to Child-Pugh B stage.

**Table 4:** BLCL staging of all patients in this study

BCLC Stage	Frequency	Percentage
B	322	85.6%
C	54	14.4%

**Table 5:** Child-Pugh staging of all patients in this study

Child-Pugh staging	Frequency	Percent
A	332	88.3
B	44	11.7

### Multivariate linear regression analysis (stepwise)

Multivariate linear regression analysis (stepwise) were used to probe the potential relationships of different factors such as hepatic artery diameter, OXA preparation time, OXA manufacturers. The results suggested that OXA preparation time (R^2^ = 0.730), OXA preparation time and hepatic artery diameter (R^2^ = 0.821), and OXA preparation time, hepatic artery diameter, and OXA manufacturers (R^2^ = 0.859) were found to be influential factors for pain scores.

## Discussion

About 90% of liver cancer recurrences after surgery occur in the liver [6]. Postoperative immunosuppression may be the cause of early recurrence [17, 18]. Furthermore, multicenter-origin liver cancer is also prone to cause the recurrence of liver cancer after surgery, which is easy to be ignored because of its small size before or during surgery [19]. Besides, tumor diameter, number, and presence or absence of microscopic tumor emboli are currently recognized as major risk factors that can affect postoperative recurrence [7]. Even if the tumor is completely removed, the risk of recurrence after surgery is still very high. Therefore, how to suppress or eliminate this intrahepatic microcarcinomas, and to eliminate the high-risk factors after surgery is an important issue to improve the tumor-free survival rate after surgery [20]. In the present study, we have focused on the analysis of influencing factors of pain induced by HAIC, and found that OXA preparation time, hepatic artery diameter, and OXA manufacturers are the potential influence factors for pain scores in HCC patients under HAIC.

Researchers have used a variety of methods to prevent recurrence of liver cancer after surgery such as super-guided percutaneous portal vein chemotherapy, intraoperative hepatic artery intubation or double hepatic artery and portal vein intubation with full implantable drug infusion devices, regular chemotherapy embolization, medical treatment and immunotherapy [21, 22]. However, no good effects were obtained. HAIC is used to deliver drugs (mainly chemotherapeutic drugs) directly to the tumor’s blood supply artery via hepatic artery intubation. For HCC, HAIC is mainly used for the treatment of advanced unresectable, transcatheter arterial chemoembolization (TACE) after recurrence and diffuse HCC patients with or without portal vein thrombosis. In theory, HAIC can directly inject a high concentration of chemotherapeutic drugs into the tumor-supplying arteries, thus improving the control effect of local liver tumors and reducing the whole body of chemotherapy drug toxicity [23, 24]. In Asia, especially in Japan and Korea, HAIC has been used as a method to improve the efficacy of advanced HCC and written into the latest Japanese HCC treatment guidelines (J-HCC 2013) [25]. Up to now, it still lacks large-scale randomized clinical trials for HAIC and requires certain operating techniques, and there is a risk of catheter-related complications. Further clinical studies are needed to confirm its effectiveness and best treatment options. In this study, it is suggested that 64.6% of patients suffer pain in HAIC.

OXA is an platinum-based chemotherapeutic agent via intravenous infusion (IV) over 2 h [26]. It was proved that OXA is effective in treating fast-growing tumors such as colorectal cancer, ovarian cancer, breast cancer [27, 28]. OXA has been reported to show adverse effects such as elevated serum aspartate aminotransferase (AST), alanine aminotransferase (ALT), bilirubin in liver [29]. This study suggested that OXA is an important factor that influences the degree of pain of HCC patients. It was speculated that there may be three reasons for the patient’s pain: 1) OXA is formulated for too long, causing oxalic acid to precipitate and stimulate blood vessels; 2) The diameter of the hepatic artery is too small. Meanwhile, insufficient blood supply to the liver leads to relatively ischemic liver and pain during the infusion of chemotherapeutic drugs.

Cancerous pain is mostly caused by tumor development and invasion. Specific pain manifests as knife-cut pain, burning pain, colic, tingling pain, radiation pain, and penetrating pain. Analgesics including opioids such as oxycodone and ibuprofen are often used in the clinic to relieve pain and supplemented with antidepressants and anticonvulsants [30]. Traditional analgesics are often not effective in the pain caused by HAIC, and large doses of strong opioids may cause serious adverse reactions such as respiratory depression. Lidocaine is often used as a local anesthetic in local anesthesia and in treating arrhythmias. Some scholars have also found that lidocaine during arterial chemoembolization can achieve analgesia and further reduce the length of hospital stay [31]. Further, other scholars suggestd that intra-arterial injection of lidocaine in TACE is more effective in reducing the incidence and severity of postoperative pain. In addition, in order to reduce the incidence of postoperative pain and the amount of postoperative analgesics, they recommend routine use of lidocaine before TACE, because patients without any pain during TACE may experience postoperative pain [32]. Our study found that lidocaine is effective in 96% (361/376) of patients during HAIC, reduces the VAS score by 85.6%, and shows no adverse reaction. The half-life of lidocaine intravenously is about 17 min, and most of it is degraded by liver microenzyme to the intermediate metabolite monoethylglycine xylene. This may also be the reason for the more sustained analgesic effect of hepatic artery perfusion with lidocaine. Due to the first-pass effect of the liver, the concentration of lidocaine in the venous blood may be low, and the effect on the heart rhythm is reduced. However, this needs to be verified by further clinical pharmacokinetic trials.

The VAS scoring method is a widely used method for measuring sensory intensity at the current clinical stage. It is worth noting that the elderly and those with mental disorders and advanced cancer patients are generally not good at using VAS evaluation. The more intuitive facial expression map will work well to evaluate the degree of pain and reduce the interference of other factors [33]. There are many reports on the clinical application of the VAS scoring system. For example, Myles et al. suggested that analgesic interventions that provide a change of 10 for the 100 mm pain VAS signify a clinically important improvement or deterioration [34]. This study applied the VAS scoring method to the assessment of pain levels in HCC patients with HAIC treatment. Based on the results of the VAS score, we implemented comprehensive pharmacological and non-pharmacological analgesic care content. The pain of cancer patients was generally relieved compared with those before the nursing intervention. Therefore, the VAS score is reasonable for assessing the pain of tumor patients. On this basis, targeted nursing intervention for patients with cancer pain can effectively relieve pain and improve the quality of life of patients.

## Conclusion

In summary, an effective way was demonstrated in this study to systematically assess and ease pain in HCC patients with HAIC treatment. OXA preparation time, hepatic artery diameter, and OXA manufacturers were the potential influence factors for pain of HCC patients under HAIC. This work presented here will provide a detailed understanding of the clinical application of HAIC in advanced HCC patients.

## Data Availability

The authors of this study agree to share the data and materials of this paper.
